# PReS-FINAL-2124: Electromyography assessment in localized scleroderma

**DOI:** 10.1186/1546-0096-11-S2-P136

**Published:** 2013-12-05

**Authors:** TP Fernandes, TD Fernandes, LFB Angulski, LAL Resende, CS Magalhaes

**Affiliations:** 1 Pediatrics, UNESP- São Paulo State University, Botucatu, Brazil; 2 Neurology, UNESP- São Paulo State University, Botucatu, Brazil

## Introduction

Localized Scleroderma (LS) has been associated with central and peripheral nervous system involvement. Facial palsy, extra-ocular movement disorders, trigeminal neuralgia and hemi-masticatory spams are described as primary neurologic involvement. Romberg hypothesized that sympathetic regulation has pathogenic relevance in facial hemiatrophy, which was reproduced experimentally by superior cervical ganglion ablation (Resende LA et al. 1991).

## Objectives

Investigate nerve conduction and muscle involvement by electromyography (EMG) in LS.

## Methods

A series of 23 LS cases with long-term follow up was retrospectively evaluated based on clinical, serological and imaging findings. Ten parents/patients agreed to participate by giving informed signed consent/ assents, being enrolled for EMG performed in *Nihon-Kohden Neuropack S1, MEB 9400 *machine. It was performed with bilateral symmetric technique, using needle electrodes for extremities. Bilateral symmetric surface quantitative electromyography (QMG) was obtained from the masticatory muscles in facial hemiatrophy/Parry-Romberg (P-R) syndrome.

## Results

A preliminary analysis of 7 electromyograms, being 5 of linear LS extremities and 2 of P-R facial muscles, is presented. Four LS had myopathic EMG pattern in muscles underlying linear streaks and 1 presented neurogenic EMG pattern. Motor and sensory nerve conduction studies of median and ulnar nerves (upper limbs) and sciatic nerve (lower limbs) resulted normal in all. Masticatory muscle testing by QMG showed reduced root mean squares and increased turns per second in the atrophic face of 2 P-R cases.

## Conclusion

There is muscle and peripheral nervous system dysfunction in LS and P-R syndrome, possibly related to inflammation and progressive soft tissue atrophy, that needs to be further explored in collective studies.

## Disclosure of interest

None declared.

